# The need for accurate estimation of sodium intake in nutritional studies

**DOI:** 10.1111/jch.14242

**Published:** 2021-03-19

**Authors:** Zeid J. Khitan, Sheena Pramod, Iheanyichukwu Ogu

**Affiliations:** ^1^ Division of Nephrology Internal Medicine Department Marshall University Joan C. Edwards School of Medicine Huntington West Virginia USA

We read with interest the study by Odili et al “Urinary sodium excretion and its association with blood pressure in Nigeria: A nationwide population survey”.^1^ We applaud the researchers' technique in obtaining a nationwide representative sample to include subjects from various geopolitical zones and different communities. We agree with the authors that such studies are highly needed and are of utmost importance in the sub‐Saharan African region especially that hypertension is a major player in cardiovascular disease outcomes and is expected to be of the salt‐sensitive type in this population. However, there are methodological limitations to this study that need to be considered while interpreting its results.

Firstly, there are inherent limitations in most epidemiological studies when addressing a nutrient or micronutrient (eg, sodium) and markers of cardiovascular disease or its outcome. Reverse causation bias precipitated by preexisting medical conditions such as hypertension and heart failure which would impact sodium and other nutrients intakes, and effect of physical activity and frailty on daily calorie intake as well as nutrients and micronutrients intake can cloud effort to establish association or causality. Adjustment for all major cardiovascular morbidities along with other markers of physical health, like the level of physical activity, would help attenuate these biases.

Secondly, estimating daily sodium intake in this study was critical to the conclusions made by the authors. In general, methods employed for estimating daily sodium intake in global nutritional studies have been heavily criticized in the past.^2^ The use of measured 24 h urine sodium is expected to be the gold standard in estimating sodium intake in dietary epidemiological studies.^3^ However, accuracy of urine collection to reflect a 24 h period is also critical to this estimation and this can usually be judged by the amount of creatinine excreted. In the study by Odili et al, 24 h urine creatinine was quite low (<10 mg/kg/24 h) when compared to the general population of diverse ethnicity making the accuracy of urine collection in question and hence the estimated daily sodium intake.^4^ Moreover, serum creatinine was not reported in their study. Nevertheless, if serum creatinine is found to be lower than expected in this study, it could have made the case for possible effects of malnutrition and poor protein intake on serum and urine creatinine although this has not been the case since serum creatinine in the sub‐Saharan African region is quite comparable to the rest of the world and generally higher in subjects from African compared with European ancestry.^5^, ^6^

Finally, with cardiovascular disease topping the list of the non‐communicable diseases in the world and knowing that populations dietary behavior is potentially modifiable,^7^ we cannot stress enough the importance of well‐designed nutritional studies to provide some answers that can help achieve better global health.

## CONFLICT OF INTEREST

The authors declare they have no competing interest with the current work.
